# Sense of School Belonging and Paediatric Illness: A Scoping Review

**DOI:** 10.5334/cie.32

**Published:** 2021-10-15

**Authors:** Lucrezia Tomberli, Enrica Ciucci

**Affiliations:** 1 University of Florence, IT

**Keywords:** hospital-based school, child with special needs, sense of school belonging, hospital teachers, school attendance

## Abstract

The experience of hospitalization leads children to move away from their everyday life, such as school attendance. Participating in school activities and relating with classmates are important experiences in children’s development and promote a general sense of school belonging.

A scoping review was conducted on the sense of school belonging (SoSB) of school-age children with medical conditions. The review concerned four specific research questions: (a) How is SoSB studied and indexed? (b) Has research on this topic changed over time? (c) What methods and techniques are used to study this topic? and (d) What role does SoSB play in the life of pupils with medical conditions? Four databases were searched: PubMed, Scopus, PsycInfo, and Education Source. The abstract and full-paper screening process identified 10 articles. A qualitative line of argument metasynthesis highlighted numerous interesting aspects: SoSB is a psychological need for pupils with a medical condition and information and communication technologies (ICT) offer an opportunity to promote SoSB and make pupils feel greater levels of well-being, less pain, and fewer negative emotions. Promoting SoSB is important for fostering a better quality of life for children with illness, helping them feel more normal and part of the class, despite not being present; hospital and regular schools should engage in creating connection opportunities for pupils with medical condition and their classes.

## Introduction

The research literature has shown an interest in the school experience of children with chronic illness. For example, children with medical conditions (a) demonstrate greater absenteeism from school and consequently are at greater risk of school dropout (Emerson et al., 2016; French et al., 2013; Shute & Walsh, 2005; Stehbens et al., 1983); (b) experience greater learning difficulties and behavioural problems, less school motivation and engagement, and lower achievement (Armstrong et al., 1999; Bonneau et al., 2011; Hay et al., 2015; Herold & Roetzheim, 1992; Pini et al., 2012). Further, pupils with chronic health issues are less involved in social relationships (Ishibashi, 2001; Leger & Campbell, 2008; Yilmaz et al., 2014) and display emotional and relational difficulties when returning to school (Harris, 2009; Katz et al., 1989, 2011; Larcombe et al., 1990; Prevatt et al., 2000; Sexson & Madan-Swain, 1993; Steinke et al., 2016; Tougas et al., 2019).

For these reasons, it is essential to promote social support during hospitalization or home care, especially from family members (Trask et al., 2003; Woodgate, 1999), peers (Ingersgaard et al., 2021; Soejima et al., 2015), and teachers (Äärelä et al., 2018; Ingersgaard et al., 2021; Lindsey, 1981; McCarthy et al., 2017). Indeed, research has established that social support is a protective factor for patients’ psycho-physical well-being; in the case of children, the support of peers (e.g., friends, classmates) seems especially important. Furthermore, participation in school life impacts the emotional and relational well-being of children and adolescents; that is, students who feel a sense of school belonging experience a general greater well-being (Arslan, 2018; Tian et al., 2016).

This review focused on the relationship between pupils with medical condition and their classmates, particularly, their sense of school belonging (SoSB). This area fits into the broader topic of “sense of belonging” (SoB), which has been studied in different populations (young people, adults, couples, etc.) (Dixon, 2018) and in different contexts (e.g., groups, school, work, sport, teams) (Carron & Chelladurai, 1981; Goodnow & Grady, 1993; Mahar et al., 1993; Walseth, 2016).

SoSB refers to a sense of belonging, relationship, and relatedness to the class and school as perceived by the individual pupil (Gowing, 2016; Libbey, 2004). In-depth studies have been conducted of the development of the terms “school belonging” (Barber & Schluterman, 2008; Kirkpatrick, 2020; Slaten et al., 2016) and “relationships to school” (Libbey, 2004). Specifically, Libbey (2004) highlighted that school-pupil relationships are classified under different labels due to the many dimensions of SoSB, including school motivation and engagement (positive orientation to school, school involvement, academic engagement, etc.), relationships with peers or teachers (school attachment, attachment to school, school bond, school connectedness, school connection, school membership, teacher attachment, teacher support), and the school context (school climate, school context, student satisfaction with school, student identification with school, etc.).

A positive SoSB correlates with greater commitment, motivation, and school achievement, as well as better mental well-being (Catalano et al., 2004; Connell & Welborn, 1991; Goodenow, 1993; Svavarsdottir, 2008; Valeski & Stipek, 2001). On the contrary, pupils who experience school disconnectedness and feel detached from the school context tend to have greater psychological and behavioural difficulties and lower academic outcomes (Murray & Greenberg, 2000; Slaten et al., 2016; Voelkl, 1997).

These findings are of particular importance for students with medical conditions, who are often left out of the class and school context due to hospitalization or home care. In order to help strengthen SoSB in pediatric populations with medical condition, it is important first to understand the state of the art and highlight gaps in the current research on the topic. This review of the relevant literature focused on four questions:

Question 1: Is the literature on SoSB well indexed and differentiated from the general social support literature, as suggested by Libbey (2004)?

Question 2: In which countries, years, and populations has the topic been studied the most? Has research on this topic changed over time?

Question 3: What methods and techniques are used to study this topic?

Question 4: What is the role of SoSB in the life of children with a medical condition and what has been done to promote SoSB?

## The Present Study

SoSB has many definitions and operationalizations making it difficult to summarize the literature on this topic. To date, most of the literature has focused on general social support and not specifically on SoSB (Libbey, 2004). For this study, we chose to conduct a scoping review to investigate how SoSB has been studied in children with medical condition in order to highlight current gaps in the research on the topic and future prospects (see Munn et al., 2018).

## Method

### Preliminary Search for Appropriate Keywords and Queries

The first step was to define the most appropriate keywords for the research questions (Siddiqi & Sharan, 2015). In order to identify the concept of SoSB, we first looked at how the pupil-class connection is defined in the literature. However, as noted by Libbey (2004), different keywords and terms have been used to indicate the topic in the literature. For example:

- The pupil’s school experience can be described using various terms: *hospital school; hospital-based school; hospital-based education; hospital teaching; normal school; traditional school; school*; *school in hospital, etc*.;
- The patient can be referred to by various more or less specific terms: *student; patient; hospitalized child; child with a chronic medical condition; child with [specific pathology]; child with special educational needs*; *child with a medical condition, etc*.

To be sure that we answered Question 1 adequately, we extended the lexicon on the subject using the thesauri of three of the four databases (PuBMed, PsycInfo, Education Source). After completing this process, we decided to use Libbey’s keywords (2004) and chose the following keywords and accompanying definitions: *school engagement* (positive orientation to school, school involvement, academic engagement), *relationships with peers or teachers* (school attachment; attachment to school; school bond; school connectedness; school connection; school membership; teacher attachment, teacher support), and *school context* (school climate, school context, student satisfaction with school, student identification with school).

### Search Strategy and Study Selection

**Figure 1:**
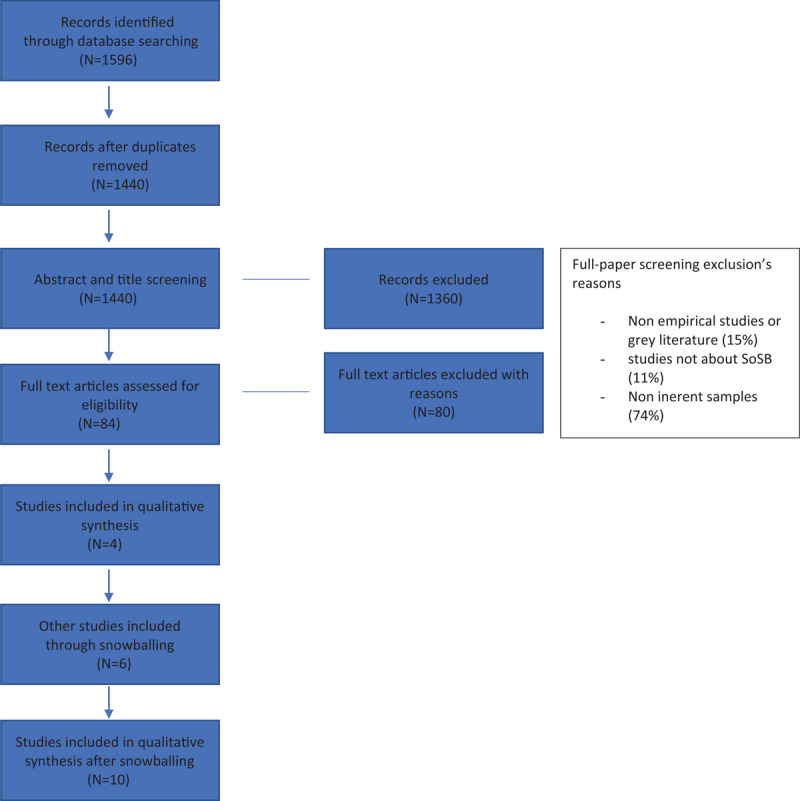
PRISMA Diagram. *Note*: Adapted from Moher et al., 2009.

The PRISMA diagram, adapted from Moher and colleagues (2009), presented in ***Figure 1*** shows the paper screening and snowballing processes conducted (Badampudi et al., 2015; Greenhalgh & Peacock, 2005; Wohlin, 2014).

- *Inclusion and exclusion criteria*: Two eligibility criteria were stipulated: (a) studies about school-age children with medical conditions frequenting hospital-based schools or homeschooling and SoSB; and (b) empirical studies. Based on these criteria, we excluded (a) non-empirical studies or grey literature (material produced outside traditional publishing and distribution channels); (b) studies not about SoSB (e.g., about general social support, sport or academic achievement); (c) different types of samples (e.g., adults, healthy children, or children with clinical conditions not attending home or hospital school). No date range was selected.
- *Databases*: Four databases were used: PubMed, Scopus, PsycInfo, and Education Source.
- *Keywords*: Starting from thesauri and Libbey’s keywords, we searched for the following terms as defined above: *school engagement, relationships with peers or teachers*, and *school context*.
- *Descriptives of the articles*: Articles are presented and summarized by (a) kind of journal, (b) country of the sample studied, (c) method, (d) disease, (e) sample description.

After screening the complete papers, 10 articles were selected as meeting the inclusion criteria (see ***Table 1***). Both authors monitored the process separately and agreed on the selected articles. The criteria for inclusion and exclusion of papers were based on the purpose of the study. The decision to eliminate grey literature arose from the need to understand what the current state of the scientific literature is.

**Table 1:** Articles Included in the Systematic Review and Descriptions.


ARTICLE	JOURNAL	COUNTRY	METHOD	DISEASE	SAMPLE DESCRIPTION

Branch-Smith, C., Shaw, T., Lin, A., Runions, K., Payne, D., Nguyen, R., Hugo, H., Balding, L., & Cross, D. (2018). Bullying and mental health amongst Australian children and young people with cystic fibrosis. *American Journal of Orthopsychiatry, 88*(4), 402–412. Scopus. *https://doi.org/10.1037/ort0000289*.	*American Journal of Orthopsychiatry*	Australia	Questionnaires Online focus group	Cystic fibrosis	*N* = 26 patients (aged 10–16) 57.7% females, 42.3% males

Ellis, S. J., Drew, D., Wakefield, C. E., Saikal, S. L., Punch, D., & Cohn, R. J. (2013). Results of a nurse-led intervention: connecting pediatric cancer patients from the hospital to the school using videoconferencing technologies. *Journal of Pediatric Oncology Nursing, 30*(6), 333–341.	*Journal of Pediatric Oncology Nursing*	Australia	Semi-structured interviews	Non-specified cancer	*N* = 3 patients (aged 8–13) 66.7% females, 33.3% males

Hopkins, L., Wadley, G., Vetere, F., Fong, M., & Green, J. (2014). Utilising technology to connect the hospital and the classroom: Maintaining connections using tablet computers and a ‘Presence’ app. *Australian Journal of Education, 58*(3), 278–296. Education Source.	*Australian Journal of Education*	Australia	Questionnaires App/web analysis Semi-structured interviews	Metabolic disease (*N* = 2), cancer (*N* = 5), cystic fibrosis (*N* = 2)	*N* = 9 patients (aged 7–12) 55.6% females, 44.4% males

Lombaert, E., Veevaete, P., Schuurman, D., Hauttekeete, L., & Valcke, M. (2006). A special tool for special children: Creating an ICT tool to fulfil the educational and social needs of long-term or chronic sick children. *Current Developments in Technology-Assisted Education, 2*, 1075–1080.	*Current Developments in Technology-Assisted Education*	Belgium	Semi-structured interviews	Not specified	*N* = 7 patients (aged 8–12) Unspecified gender

Maor, D., & Mitchem, K. (2020). Hospitalized Adolescents’ Use of Mobile Technologies for Learning, Communication, and Well-Being. *Journal of Adolescent Research, 35*(2), 225–247. *https://doi.org/10.1177/0743558417753953*	*Journal of Adolescent Research*	Australia	In-depth interviews	Non-specified disease	*N* = 18 patients (aged 12–18) Unspecified gender

Vetere, F., Green, J., Nisselle, A., Dang, X. T., Zazryn, T., & Deng, P. P. (2012). Inclusion during school absence: Using ambient technology to create a classroom presence for hospitalised children. *Telecommunications Journal of Australia, 62*. Retrieved from *http://doi.org/10.7790/tja.v62i5.353*	*Telecommunications Journal of Australia*	Australia	App/web analysis Systematic observation Focus group Semi-structured interviews	Non-specified disease	*N* = 4 patients (aged 9–10) 75% females, 25% males

Weibel, M., Nielsen, M. K. F., Topperzer, M. K., Hammer, N. M., Møller, S. W., Schmiegelow, K., & Bækgaard Larsen, H. (2020). Back to school with telepresence robot technology: A qualitative pilot study about how telepresence robots help school-aged children and adolescents with cancer to remain socially and academically connected with their school classes during treatment. *Nursing Open*. *https://doi.org/10.1002/nop2.471*	*Nursing Open*	Denmark	Participated observation Focus group Semi-structured interviews	Cancer	*N* = 3 (school age, not clearly specified)

Weiss, P. L. T., Whiteley, C. P., Treviranus, J., & Fels, D. I. (2001). PEBBLES: A personal technology for meeting educational, social and emotional needs of hospitalised children. *Personal and Ubiquitous Computing, 5*(3), 157–168.	*Personal and Ubiquitous Computing*	Canada	App/web analysis	Chronic kidney disease	*N* = 1 (case study)

Zhu, C., & Van Winkel, L. (2015). Using an ICT tool as a solution for the educational and social needs of long-term sick adolescents. *Technology, Pedagogy and Education, 24*, 231–245. doi:10.1080/1475939X.2013.856339	*Technology, Pedagogy and Education*	Belgium	Questionnaires Semi-structured interviews	Non-specified disease reported for 56 students participating in quantitative research; 8 children participating in qualitative research with: fibromyalgia (*N* = 1), vascular problems (*N* = 1), Hodgkin lymphoma (*N* = 1), transplant (*N* = 1), depressive symptoms (*N* = 1), autoimmune disease (*N* = 1), other non-specified pathologies (*N* = 2)	*N* = 56 patients participating in the quantitative part of the study (aged 12–19) *N* = 8 participants of the qualitative part (aged 12–19) 64% females, 36% males

Zhu, C., & Van Winkel, L. (2016). A virtual learning environment for the continuation of education and its relationship with the mental well-being of chronically ill adolescents. *Educational Psychology*, 36(8), 1429–1442.	*Educational Psychology*	Belgium	Questionnaires In-depth interview	Non-specified disease	*N* = 28 (aged 10–18)68% males, 64% females


To analyze the results, we used a qualitative line, or argument metasynthesis; that is, a conceptual synthesis (Attree, 2006; Montù, 2015), which can be conducted with various types of software (we chose QCAmap – Qualitative Content Analysis software, which is available at *https://www.qcamap.org/*).

Therefore, we carried out thematic analysis starting from the contents of the articles. Thematic analysis consists of attributing meaning labels (themes) starting from the contents of a text, narration, article, etc., and allows identification of the topics (themes) treated in a given text (Attree, 2006; Montù, 2015).

## Results

The results are summarized below in response to the four research questions underlying the study.

### Question 1: Is the literature on SoSB well indexed and differentiated from the general social support literature?

The keywords used in the selected articles make it clear that at present the literature on the subject is still similar to that on general social support, even though SoSB is a well-defined construct (Libbey, 2004) distinctly investigated in articles included in the review. In fact, as illustrated in ***Table 2***, the most frequently used keywords in the selected articles were *child/adolescent, cancer* or *chronic illness, special educational needs*, and *ICT*. The articles were not indexed under the topic of SoSB.

**Table 2:** Keywords Used for Indexing.


ARTICLE	ARTICLE KEYWORDS

Branch-Smith et al., 2018	*Adolescents; bullying; children; chronic condition; cystic fibrosis*

Ellis et al., 2013	*Psychology; pediatric oncology; social networking; intervention studies; education*

Hopkins et al., 2014	*Chronic illness; primary school students; student engagement; information and communication technology in education; tablet computers; attendance*

Lombaert et al., 2006	*Special educational needs; computer-assisted learning*

Maor & Mitchem, 2020	*Hospitalized adolescents; mobile technologies; learning; communication; well-being*

Vetere et al., 2012	*Schools; child; chronic illness*

Weibel et al., 2020	*Cancer; childhood illness; education; school nursing; technology*

Weiss et al., 2001	*Hospitalised children; personal technology; telepresence; video-conferencing*

Zhu & Van Winkel, 2015	*ICT tool; educational needs; social needs; long-term sick adolescents*

Zhu & Van Winkel, 2016	*Virtual learning environment; educational needs; social needs; mental well-being; chronically sick adolescents*


No differences emerged in the use of the keywords depending on the year of publication; in fact, it appears that varied and undifferentiated keywords are generally used for indexing articles. Also, it seems that, in general, the literature is indexed around three areas: paediatric disease, the need for relationships, and ICT.

### Question 2: In which countries, years, and populations has the topic been studied the most? Has research on this topic changed over time?

#### Types of Research on the Topic

The articles included in the review were published in various fields such as psychology, psychiatry, nursing, education, and computer sciences and were conducted in different countries: Australia (*N* = 5; Branch-Smith et al., 2018; Ellis et al., 2013; Hopkins et al., 2014; Maor & Mitchem, 2020; Vetere et al., 2012); Belgium (*N* = 3; Lombaert et al., 2006; Zhu & Van Vinkel, 2015, 2016); Canada (*N* = 1, Weiss et al., 2001); and Denmark (*N* = 1, Weibel et al., 2020).

The selected studies were published between 2001 and 2020. Since we did not set range limits on the year of the articles, it seems that SoSB has been a topic of particular research interest in the last 20 years: indeed, the previous literature focused exclusively on the issue of general social support.

#### Types of Illnesses Included in the Selected Articles

The illnesses studied in the selected articles included chronic kidney disease (*N* = 1), complex transplants (*N* = 1), vascular problems (*N* = 1), juvenile fibromyalgia (*N* = 1), metabolic disorders (*N* = 2), cancer, (*N* = 10), cystic fibrosis (*N* = 28), and unspecified illnesses (*N* = 111). Only one of the articles was a case study; the others included samples ranging from a minimum of 3 (Ellis et al., 2013) to a maximum of 56 participants (Zhu & Van Vinkel, 2015) (the average number of participants was 29.5). As required by the inclusion criteria, all the children in the studies were school age (42% aged 7 to 13 years; 46% aged 14 to 16; 12% aged 17 to 18).

### Question 3: What methods and techniques are used to study this topic?

A wide variety of research methodologies were identified, ranging from the use of questionnaires, surveys, apps, and web analysis, at a quantitative level, to the use of semi-structured interviews, in-depth interviews, focus groups and qualitative observations, at a qualitative level. Half of the articles used mixed methods (see ***Table 1***). Choice of method did not depend on the year of publication of the articles. Some studies preferred online modalities because of the children’s medical condition (20%). Concerning the articles that used mixed methods, only 42% (Branch-Smith et al., 2018), 14% of the total participants (Zhu & Van Vinkel, 2015), participated in the qualitative part, because it was considered more emotionally engaging, according to the authors’ hypotheses.

### Question 4: What is the role of SoSB in the life of children with a medical condition and what has been done to promote SoSB?

Three main topics were found related to SoSB:

1. SoSB as a psychological need for pupils with a medical condition (Branch-Smith et al., 2018; Lombaert et al., 2006; Zhu & Van Vinkel, 2015).
2. Opportunities for ICT to promote SoSB (Hopkins et al., 2014; Maor & Mitchem, 2020; Weibel et al., 2020; Zhu & Van Winkel, 2016).
3. Effectiveness and psychological outcomes of interventions promoting SoSB (Ellis et al., 2013; Vetere et al., 2012; Weibel et al., 2020; Weiss et al., 2001; Zhu & Van Winkel, 2016).

No information was found regarding body image, even though the literature shows that body issues matter a lot to children and adolescents in hospital settings; in fact, studies have found that “showing themselves” to classmates through ICT can be very stressful for pupils with a medical condition because of the appearance sometimes caused by their therapies (e.g., hair loss, swelling, scars) (Fan & Eiser, 2009; Larouche & Chin-Peuckert, 2006; Pendley et al., 1997).

**Figure 2:**
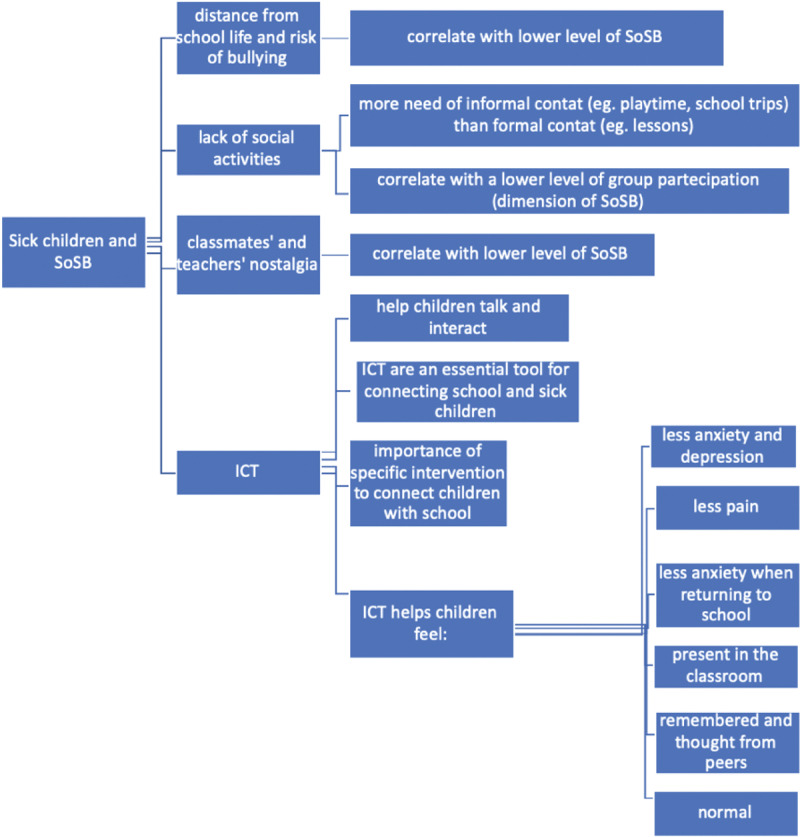
Children With a Medical Condition and SoSB: Correlated Dimensions, Emotions, and Cognitions.

The qualitative line of argument metasynthesis allowed us to highlight the three noteworthy findings (see ***Figure 2***).

### SoSB as a Psychological Need for Pupils With a Medical Condition

Sometimes, children with chronic health issues experience anxiety or depression and are victims of bullying (Branch-Smith et al., 2018). Pupils with cystic fibrosis, for example, reported being victims of direct (52.9%) and indirect (verbal, 58.8%; social exclusion, 23.5%) bullying (Branch-Smith et al., 2018); experiences of bullying correlated with lower levels of SoSB in pupils with chronic disease. Moreover, teenagers experienced more bullying and a greater extent of school disconnectedness than younger children. Among primary-school children with cystic fibrosis, 34.62% felt socially supported; students who were hospitalized for a long time or who, due to their illness, were often absent from school and did not take part in shared social activities, such as group activities, games, or trips, reported a lower sense of participation (Lombaert et al., 2006). In addition, pupils with a medical condition experienced a sense of nostalgia for their classmates and teachers (Lombaert et al., 2006; Zhu & Van Winkel, 2015), whom they perceived as distant and not very participatory during their hospitalization. Zhu and Van Winkel (2015) also noted that for pupils with a medical condition, informal contacts with classmates (e.g., playtime, recreation) were more significant than sharing class activities (e.g., doing tasks, keeping up with the programme, following lessons); the reviewed programmes seemed to foster a sense of “normality” in the pupils with chronic health issues (Ellis et al., 2013; Weibel et al., 2020).

Lombaert and colleagues (2006) emphasized that new technologies could improve the connection between children with a medical condition and regular school. Similarly, Zhu and Van Winkel (2016) found that 60% of the participants in their study found school fundamental in maintaining contacts with their peers.

#### Opportunities of ICT for Promoting SoSB

Thirty percent of the selected articles (Branch-Smith et al., 2018; Lombaert et al., 2006; Maor & Mitchem, 2020) investigated the opinions of children regarding the use of ICT to connect them to their class and the importance of being connected. By comparison, 70% of the articles specifically described interventions using ICT to connect hospital/home and school. The interventions are presented in ***Table 3***.

**Table 3:** Description of the Projects in the Articles.


ARTICLE	

Ellis et al. (2013)	**Connectivity Project** The Connectivity Project was conducted at the Sydney Children’s Hospital (Australia); it had a variable duration and structure. Sometimes, the children participated for a few days, at other times for a few months if the pathology forced them to stay in the hospital for long periods. The connection sessions lasted approximately one hour. The programme allowed children with a medical condition to connect with school or with their homes. The aim of the programme was to improve the connections between the children’s various contexts (hospital, home, school).

Hopkins et al. (2014)	**Presence App** This app enables patients, family members, and classmates to connect with each other. It allows taking, archiving, and sharing photographs; it is also possible to send symbolic “messages” using colour as a vehicle. This means that the participants (e.g., the child with a medical condition) can decide to “colour” the app orange or blue, and all those connected would see the interface change colour. The purpose is to foster the sense of “presence” of the child admitted to the class where the device is inserted. Through the app, the child can see which lessons his or her classmates are following at school. The Presence app was inspired by the Ambient Orb proposed in other studies (see Vetere et al., 2012). Unlike the Ambient Orb, however, the Presence app is bidirectional: It not only allows the child to communicate with the class, but also allows the class to communicate with the child.

Vetere et al. (2012)	**Ambient Orb** The technological connection environment consists of a sort of “sphere” with a human-like face and a LED inside that allows the sphere to change colour. The sphere, which was inserted in each class of the pupil with a medical condition, does not allow a great deal of interaction (photos, audio, video). Each sphere had a wireless connection to a nearby laptop. The child in the hospital could use a computer to check the colour of the sphere placed in the classroom in order to signal his or her presence to classmates and teachers. This was the only function of the tool.

Weibel et al. (2020)	**AV1, No-Isolation** AV1, built by No-Isolation, is a robot connected to an app that can be accessed by phone; the device was designed to connect children with cancer with their class.

Weiss et al. (2001)	**P.E.B.B.L.E.S** PEBBLES (*Providing Education By Bringing Learning Environments to Students*) is a video-conferencing device designed to connect pupils with a medical condition to their regular school. Part of the PEBBLES system was set up in the classroom and part in the child’s ward; in the classroom, the device was supported by a robot so that it could be moved easily if the classmates moved (e.g., from one classroom to another one). PEBBLES allows children to share images and audio by video-conferencing, so that they can follow the lessons. In the study, PEBBLES was used three times a week during the morning with one child. Both the pupil in the hospital and the class started lessons in sync at 9.00 a.m.; the child was accompanied by the hospital teacher for the whole period that PEBBLES was used.

Zhu & Van Winkel (2015)	**BEDNET** The participants used remote connection tools for three months up to three years, in the case of long-term hospitalized children. A device made available by Bednet VSW was used; the device made it possible to connect the hospitalized pupil to the school. It was a bidirectional device with synchronous audio/video in order to allow the connection in “presence” mode between the pupil with medical condition and the classroom. It consisted of various components: a webcam, a blackboard, a sound button (to report something to the class), a drive (to post photos, images, documents), a document scanner and a class agenda (a sort of electronic register). This tool allowed pupils with chronic health issues to follow the lesson, raise their hands, answer questions, ask the teacher for attention, take pictures on the blackboard, etc., in sync with what the class was doing.

Zhu & Van Winkel (2016)	**Virtual Learning Environment** A virtual learning environment (VLE) was created allowing synchronous communication between the pupil with a medical condition and the other members of the class. The VLE offered various functions: a virtual whiteboard, a virtual library, buttons to request attention, a webcam, a scanner, and the ability to send documents and share photos.


Hopkins and colleagues (2014) and Maor and Mitchem (2020) noted that ICT is useful for connecting pupils with medical conditions with their classmates, because it helps them talk to and interact with their peers, thereby reducing social isolation (Maor & Mitchem, 2020) and making pupils feel less alone, even though pupils report that ICT cannot replace face-to-face interactions because it is less interactive. Further, Weibel and colleagues (2020) and Zhu and Van Winkel (2016) stressed that connecting the children with their class favoured better psychological outcomes. That is, technologies such as ICT are regarded as somehow therapeutic (Maor & Mitchem, 2020), because they reduce perceived pain and fosters a greater sense of presence and SoSB, and a greater general perception of social support (Maor & Mitchem, 2020; Weibel et al., 2020; Zhu & Van Winkel, 2016). Studies have also shown that the presence of robots in the classrooms fosters a greater sense of connection with peers (Zhu & Van Winkel, 2016) and greater inclusion in regular classroom activities (Weibel et al., 2020).

However, the authors also pointed to limitations in the use of ICT, although this was not specifically one of their goals. For example, children have reported problems with Wi-Fi connections (Hopkins et al., 2014) and a general difficulty in remaining attentive in blended learning due to drug therapies (Maor & Mitchem, 2020). Further, teachers do not usually use ICT or the robot in the classroom is ignored and the pupils’ presence in the class, therefore, is not felt (Weibel et al., 2020).

#### Effectiveness and Psychological Outcomes of Interventions Promoting SoSB

All the selected articles evaluated programmes that promoted SoSB as effective (Ellis et al., 2013; Hopkins et al., 2014; Vetere et al., 2012; Weibel et al., 2020; Weiss et al., 2001; Zhu & Van Winkel, 2015, 2016). The interventions promoting SoSB were mediated by apps, robots, or other technological devices connecting the children with the classroom. The robots or other ICT were physically positioned in the classroom to represent the missing child, who followed the lessons simultaneously from home or the hospital.

The programmes were particularly useful for promoting a hospital-school connection (Ellis et al., 2013; Weibel et al., 2020; Zhu & Van Winkel, 2016), making the children feel included and remembered by their classmates (Vetere et al., 2012). That is, the programmes made the children feel present and as if they were participating in the classroom because the presence of a robot/app (Vetere et al., 2012; Weibel et al., 2020; Weiss et al., 2001; Zhu & Van Winkel, 2016). Zhu and Van Winkel (2016) found that 28.57% of the participants reported that participation in digital programmes led to lower anxiety levels when students returned to school, after previously experiencing worry and agitation (see ***Figure 2***).

However, using a robot or an app to connect the children with traditional school (Hopkins et al., 2014; Weibel et al., 2020) is not sufficient to make the children feel present and included in the class if not combined with a parallel didactic activity and/or shared laboratory. That is, if the children cannot actively participate in the class activities, their presence in the classroom ends up being a formality and often the children are ignored by their classmates and teachers.

## Discussion

As illustrated, the literature on school belonging is still scarce and difficult to put together in a corpus; in fact, a variety of keywords are used to index articles on the topic, despite the dedicated specialized lexicon to indicate SoSB (Libbey, 2004). The articles included in the current review were published in journals representing a range of disciplines (psychology and psychiatry, nursing, education, computer sciences), thus highlighting the multidisciplinary nature of the topic, which requires exploration from several points of view. Most of the studies were conducted in Australia (*N* = 5) and Belgium (*N* = 3). This is not surprising, considering that there is the Royal Children’s Hospital Education Institute in Australia (Melbourne) that aims to connect connect students with a medical condition with traditional schools and that Belgium is investing heavily in research on the subject. Our findings show that SoSB is considered a fundamental psychological need for pupils with chronic health issues and that technologies can represent a good way to connect children with chronic health issues with their class.

This scoping review is not without limitations; the selected studies concerned different medical conditions and were conducted in different countries. An additional aspect to study would be whether there are any differences in SoSB as perceived by children with malignant vs. benign medical conditions or if there are any differences depending on the organization of the different schools in the hospital. At the moment, it is not possible to examine this aspect further.

In the future, it would also be interesting to investigate the role of body image related to the use of technologies with pupils with chronic health issues in order to understand if the reported difficulty in “showing themselves” to their peers has not emerged because it is not actually present or because it has not been investigated by the authors of the selected studies. It would also be interesting to examine the role of hospital schools in promoting SoSB. For example, what is the role of hospital teachers? Do pupils feel SoSB even with hospital-based schools? Are there any differences between hospital-based school SoSB and homeschooling SoSB? To date, the literature only seems to focus on the children’s sense of belonging to regular school, but it would be stimulating to understand if it would be useful also to encourage SoSB with hospital teachers or other pupils who make up the school “class” in the hospital.

## Conclusions

Promoting SoSB plays an important role in fostering a better quality of life for pupils with a medical condition in order to help them feel more normal and part of their class, despite the experience of prolonged hospitalization and/or isolation from their peers.

Based on our review, we recommend that hospital and regular schools engage in creating opportunities for connections between pupils with chronic health issues and their classes, both from a didactic (continuity in learning) and a relational point of view (formal and informal contacts). More specifically, we believe that an intervention to promote SoSB should take into account (a) the presence or absence of suitable technologies within the regular school necessary to connect the child with chronic health issues; (b) the user-friendliness of technologies so that their use in school is feasible; (c) the fact that technology alone is not enough. As the review showed, children feel connected when they actively participate in school activities, not when they just observe them; and (d) pupils’ opinion about being connected to the class through appropriate technologies.

This scoping review found studies that carried out SoSB promotion projects with the use of fairly advanced technologies, such as AV-1 (Weibel et al., 2020) or the Presence app (Hopkins et al., 2014); however, technologies like these are often expensive and not usable by all children in paediatric hospitals. For this reason, we believe that it is necessary to find ways that allow a greater number of children to connect with the class; for example, through sharing tools (e.g., Google Drive, Edmodo) or Skype. Being connected with their school is a basic need for children’s quality of life. Hence, both regular and hospital schools should strive to foster opportunities to connect the children and help them feel part of the class as much as possible.

Finally, it would be interesting to investigate – also through a systematic map (Cooper, 2016; Grant et al., 2009; James et al., 2016) – the research into the topic of social support in the paediatric population with a medical condition (e.g., school activities, sports, friendships, specialist summer camps). Findings from such research would allow us to understand why, as yet, there seems to be no clear definition of the specific theme of SoSB in this population despite the centrality and importance of the topic.
